# Diagnosis, Treatment, and Potential Complications of Triplane Ankle Fractures in Pediatric Patients: A Systematic Review

**DOI:** 10.3390/jcm14051578

**Published:** 2025-02-26

**Authors:** Grayson M. Talaski, Bshara Sleem, Emily J. Luo, Julia Ralph, Lulla Kiwinda, Conor N. O’Neill, Kempland C. Walley, Albert T. Anastasio, Brian C. Lau

**Affiliations:** 1 Department of Orthopedics and Rehabilitation, University of Iowa, Iowa City, IA 52242, USA; 2 College of Medicine, American University of Beirut, Beirut 1107, Lebanon; bms26@mail.aub.edu; 3 Department of Orthopaedic Surgery, Duke University, Durham, NC 27710, USA; emily.luo@duke.edu (E.J.L.); julia.ralph@duke.edu (J.R.); lulla.kiwinda@duke.edu (L.K.); conor.n.oneill@duke.edu (C.N.O.); albert.anastasio@duke.edu (A.T.A.); brian.lau@duke.edu (B.C.L.); 4 Department of Orthopaedic Surgery, University of Michigan, Ann Arbor, MI 48109, USA; kcwalley@med.umich.edu

**Keywords:** triplane ankle fracture, pediatric, systematic review, surgical outcomes

## Abstract

**Background/Objectives:** Triplane ankle fractures represent a complex fracture type in pediatric patients. These fractures can prove challenging due to the involvement of multiple fracture planes and variations in segment fragmentation. With increasing literature pertaining to the treatment of triplane fractures and the limitations of previous systematic reviews, the aim of this review is to summarize recent data on the diagnosis, treatment, and complications of pediatric triplane ankle fractures. **Methods:** This systematic review was conducted following PRISMA guidelines and searched five major databases up to July 2024. The inclusion criteria focused on observational studies and randomized controlled trials in pediatric triplane fractures. Case reports, cadaveric studies, and systematic reviews were excluded. Articles were screened and graded using the MINORS tool for quality assessment. Data were extracted on demographics, fracture types, treatment approaches, and outcomes. **Results:** A total of 34 studies met the inclusion criteria. The use of CT scans in combination with radiographs was common, and two-part fractures were the most frequently observed type. Surgical treatment, particularly open reduction with internal fixation, was preferred, while conservative surgical treatment remains under-reported. Complications were minimal, with limb length discrepancy being the most common. **Conclusions:** This review highlights the increased use of CT for diagnosing triplane fractures and the preference of certain surgical interventions. Conservative treatment approaches, though less studied, may offer alternatives in moderate cases. This review emphasizes the need for further research on conservative treatment outcomes, longer follow-ups, and randomized controlled trials to refine treatment strategies for this complex fracture pattern.

## 1. Introduction

As triplane ankle fractures represent the second most common fracture type in pediatric patients [1], with some studies even listing it as the most common [2], it is crucial to understand the treatment and complications associated with these injuries. These fractures occur during the early stages of growth plate closure, a period when the physis is partially fused but not yet fully ossified [3]. This incomplete closure creates a structural vulnerability, allowing fractures to extend across the sagittal, axial, and coronal planes.

**Figure 1:**
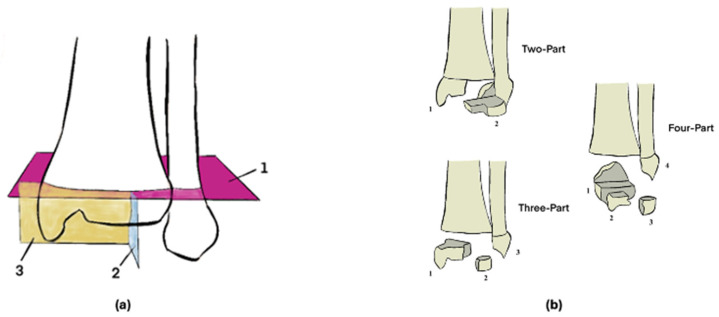
Different anatomic planes impacted by triplane fractures (**a**) and the different common fracture patterns (**b**). In (**b**), two-, three-, and four-part fractures are described, with each fracture’s various fragments labeled.

Triplane ankle fractures, which occur to the distal tibia epiphysis in pediatric patients, represent a challenging injury pattern for orthopedic surgeons. Triplane fractures represent 5% to 10% of pediatric intra-articular ankle injuries and typically occur in children aged 10 to 17 years [4]. While distal tibia epiphysis fractures can be classified according to the Salter–Harris classification [5], triplane ankle fractures are difficult to classify according to this system for a variety of reasons. To begin with, triplane fractures occur in the sagittal, axial, and coronal planes, with some variations including two-, three-, and four-part fractures, involvement of the medial or lateral regions, and configurations that can be either intra-articular or extra-articular (Figure 1) [6]. Furthermore, each epiphysis differs morphologically, in addition to in its location, weight-bearing function, and vulnerability to injury [7]. With nearly all major ankle ligamentous structures directly connecting to the distal tibial and fibular epiphyses, triplane ankle fractures are detrimental to global ankle stability and function. In pediatric patients, the lateral ankle ligamentous complex is much stronger relative to the open physis, potentially subjecting pediatric patients to growth arrest and the development of future deformity in the setting of injury about the ankle [8,9,10].

While previous systematic reviews and meta-analyses have attempted to describe triplane ankle fractures, it has been nearly five years since the last comprehensive review was published [11]. Furthermore, this review was limited in the included articles, as there has been a roughly 400% increase in the number of recently published articles describing patients with triplane ankle fractures. Due to the high prevalence of this injury pattern, we believe it is crucial that orthopedic surgeons have access to the most recent, inclusive, and comprehensive review to optimally treat and diagnose their pediatric patients. Therefore, the primary aim of this systematic review was to summarize all the data related to diagnosis, treatment, and subsequent complications.

## 2. Materials and Methods

### 2.1. Study Creation and Initial Search

This systematic review was conducted in accordance with the Preferred Reporting Items for Systematic Reviews and Meta-Analyses (PRISMA) guidelines [12]. The review protocol was not registered before its completion. The initial literature search was carried out across five databases—PubMed, CINAHL, MEDLINE, EMBASE, and Web of Science—encompassing all records retrieved by the search algorithm up to 22 July 2024. The search algorithm used in this study was (triplane fracture OR triplane fractures OR tibial triplane fracture OR triplane ankle fracture OR triplane ankle fractures OR Salter–Harris type IV fracture OR Salter–Harris Type IV fractures).

### 2.2. Eligibility Criteria

The inclusion criteria were articles that examined pediatric triplane fractures, observational studies, randomized controlled trials, articles in English, and articles with a full text. The exclusion criteria were articles that did not examine triplane fractures, articles not in English, those without a full text, case reports, cadaveric studies, and systematic reviews.

### 2.3. Study Definitions

Given that several studies included both isolated triplane fractures and cases involving additional ankle injuries, it was necessary to establish criteria to define the severity of triplane fractures in this review. Triplane fractures are categorized based on the number of fracture fragments and the planes involved. They can be classified into two-part, three-part, or four-part fractures, depending on the extent of fragmentation [13]. Two-part fractures typically involve only the sagittal and coronal planes, while three-part fractures include an additional transverse component. Four-part fractures, the most complex, extend across all three planes and often involve significant articular displacement [13]. Triplane fractures are assessed using parameters such as the specific type of triplane fracture, its location, and the degree of displacement. A triplane fracture displacement of more than 2 mm, particularly in the articular surface, is considered significant and typically necessitates surgical intervention to restore proper joint congruity and prevent long-term complications [6].

### 2.4. Article Screening Process

Following the search of the five databases using the specified algorithm, all the retrieved articles were imported into Rayyan.ai, a widely used online software for systematic review article screening [14]. Duplicate articles were initially removed manually, and the remaining articles were then screened by title and abstract according to the inclusion and exclusion criteria. The screening process was conducted by two authors. After the title and abstract screening, the full texts of the remaining articles were reviewed to determine final inclusion. All conflicts were resolved by discussion amongst the author group.

### 2.5. Data Extraction

Data extraction was performed by a single author. Data extracted from the included articles included first author, year of publication, study type, number of patients, location and type of triplane fracture, cause of injury, imaging modality (including weightbearing radiograph and computed tomography), management approach, follow-up duration, and reported outcomes, such as complications, union status, functional scores (including the American Orthopedic Foot and Ankle Society (AOFAS) Ankle–Hindfoot Scale and the modified Weber–Baker score), and additional outcomes.

### 2.6. Article Quality Grading

All observational studies were evaluated using the Methodological Index for Non-Randomized Studies (MINORS), a tool frequently applied in systematic reviews and meta-analyses [15]. The MINORS scale distinguishes between comparative and non-comparative studies, scoring comparative studies out of 24 points and non-comparative studies out of 16 points. Each item on the MINORS scale is rated from 0 to 2 points, reflecting the quality of the study. All grading was completed by two authors.

### 2.7. Statistical Analysis

The analysis for this systematic review was conducted using R, version 4.3.0 (R Foundation for Statistical Computing, Vienna, Austria). Descriptive statistics, including means and frequencies, were calculated, and frequency-weighted means were used to present the data. Due to the heterogeneity of the included studies, a narrative approach with qualitative statistics was utilized for the systematic review, as meta-analysis was not possible.

## 3. Results

### 3.1. Search Results

**Figure 2:**
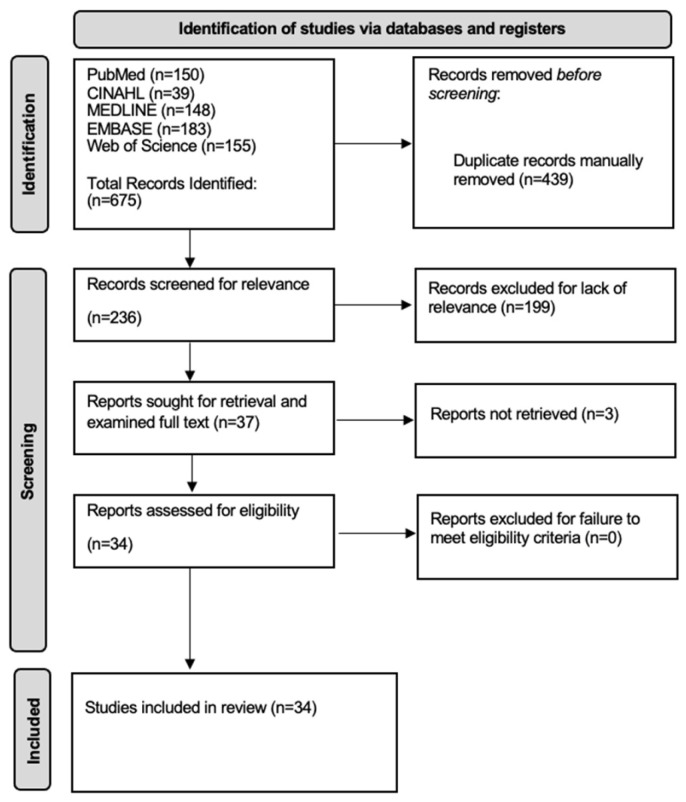
Preferred Reporting Items for Systematic Reviews and Meta-Analyses (PRISMA) diagram.

In total, 34 articles met the eligibility criteria for inclusion in this systematic review from 675 articles initially retrieved from the five databases [16,17,18,19,20,21,22,23,24,25,26,27,28,29,30,31,32,33,34,35,36,37,38,39,40,41,42,43,44,45,46,47,48,49]. All 34 articles were initially found on the five databases, and no additional articles were included from further reference searches of the included articles. Refer to Figure 2 for the PRISMA diagram outlining the search process for this systematic review.

### 3.2. Article Quality Results

All 34 included articles were evaluated using the MINORS scale, reflecting the observational and non-comparative nature of the studies. The average MINORS score across all the included articles (n = 34) was 8.65 ± 1.13 points, with scores ranging from 7.0 to 11.0 points. For the detailed MINORS grading of each article, refer to Table 1.

### 3.3. General Patient Demographics

A total of 830 patients with triplane fractures were included in this systematic review, with a frequency-weighted mean age of 13.27 ± 1.09 years (n = 810; 97.1% of studies reported). Nearly all studies (n = 33; 97.1%) reported on the percentage of female patients, with a weighted average of 41.18%. There were 205 cases of right ankle fractures and 178 cases of left ankle fractures (16 (47.1%) of the included studies reported). The mean follow-up duration reported across the studies was 30.62 months, with follow-up data available from 82.4% of the studies. Detailed information on study-specific demographics and patient characteristics can be found in Table 2.

### 3.4. Diagnostic Approaches and Common Injury Patterns

With regards to the imaging modalities used for the diagnosis of triplane fractures, 30 out of 34 studies used CT scans, while radiographs were employed in 27 studies. Additionally, tomography was used in eight studies, MRI scans in three studies, intraoperative fluoroscopy in two studies, and scanograms in one study. Of the included studies, 27 employed a combination of at least two diagnostic imaging modalities. As for the causes of fracture, injury due to sports activities (n = 127 patients; 15.3% of included patients) was one of the most common, followed by falls/slips (n = 77 patients; 9.3% of included patients), and motor vehicle accidents (n = 60 patients; 7.2% of included patients). However, most fractures had undefined etiologies (n = 512 patients; 61.7% of included patients). In terms of fracture morphology, 358 (43.1%) patients had two-part fractures, whereas 172 (20.7%) had three-part fractures.

Park et al. (2019) reported that 15 (71.43%) of their patients had fibular fractures accompanying their triplane fractures [39]. Overall, 144 (17.3%) patients had an associated fibular fracture (55.9% of studies reported). As for radiographic measurement outcomes, radiographic analysis postoperatively was under-reported. In those studies that did report postoperative radiographic analysis, 11 (1.3%) patients had a residual postoperative displacement greater than 2 mm (8.82% of studies reported). In addition, the weighted mean step-off was 0.33 (SD = 0.52; 5.88% of studies reported). Further details on diagnostic modalities, injury mechanisms, and fracture patterns can be found in Table 3.

### 3.5. Treatment and Additional Outcomes

Of the 34 studies, 31 described management of triplane fractures. The most common surgical treatment was open reduction with or without internal fixation (n = 273 patients; 32.9% of the included patients; 91.2% of studies reported), followed by closed reduction with or without percutaneous fixation (n = 202 patients; 24.3% of the included patients; 91.2% of studies reported). A total of 393 patients (47.3%) demonstrated complete union at final follow-up (55.9% of studies reported). In the studies included, the most utilized postoperative scoring scale was the modified Weber–Baker score (29.41% of studies reported), with 194 (out of 213; 91%) of patients reporting a score of “Excellent”. Detailed results pertaining to the treatments, additional outcomes, and additional scales can be found in Table 4.

### 3.6. Complications

Most studies (20 studies; 58.82%) reported complications. Leg length discrepancy was the most reported complication (10 patients; 1.2% of included patients). Ma et al. reported four cases of implant-related complications (such as hardware loosening or breakage) and three cases of infection-related complications [35]. Data on decreased range of motion (ROM) were limited. Rapariz et al. reported four cases of a postoperative decrease in ROM [41]. Aslam et al. reported no complications following the treatment of triplane fractures in their cohort of three patients [16]. They specifically mentioned that no patients experienced any issues related to the surgical procedure, recovery, or rehabilitation. Similarly, Tan et al. (2013) observed no surgical complications. In total, five studies reported zero complications. Finally, twelve patients (1.4%) had premature physis closure (PPC) (20.6% of studies reported) [44]. Refer to Table 5 for more information on complications from individual articles included in this study.

## 4. Discussion

In this systematic review, the diagnosis, treatment, and complications pertaining to triplane ankle fractures were studied. One previous systematic review has been published regarding triplane ankle fractures, but it was limited to imaging modalities and management strategies [11]. Our review offers a broader scope to include important pre-management factors, such as the cause of injury, as well as potential long-term postoperative complications. Furthermore, the present review includes a large cohort of 830 patients, compared to the previous review’s inclusion of only 203 patients. As triplane ankle fractures represent the second-most common fracture type in skeletally immature individuals [1], we believe it is crucial to study not only a large group of patients but also to summarize the most recent advancements pertaining to the treatment of this patient cohort.

Similar to previous reviews [11], over 60% of the included patients were male. However, our review studied a broader distribution of fracture types, with 43.1%, 20.7%, and 2% of patients having two-, three-, or four-part fractures, respectively. Compared to previous reviews, where the fracture type was heavily skewed towards two-part fractures (68%) [11], our review may provide a more comprehensive understanding of the clinical outcomes associated with triplane fractures, regardless of fracture type. However, only 67.6% of studies reported the fracture type, which makes the interpretation of results specific to each fracture type difficult. Compared to previous reviews, the use of computed tomography (CT) combined with radiography for fracture diagnosis has grown in popularity, with up to over 90% of the included articles describing the use of this imaging modality combination. This shift will likely impact surgeon decision making, as certain studies have demonstrated that by adding a CT evaluation, significantly more patients who were assigned non-operative management were reassigned to operative treatment [50]. In addition to CT offering a three-dimensional view, compared to two-dimensional radiographs, this change in decision making is likely a result of a more accurate understanding of fracture displacement with CT [48]. While radiographs present numerous benefits, such as lower radiation doses and costs [51], technologies such as cone-beam CT present an appealing alternative [52]. With the increase in popularity of cone-beam CT in the diagnosis of numerous orthopedic pathologies [53,54,55], we anticipate a similar trend for complex deformities such as triplane fractures.

Overall, the risk of postoperative complications was low. Across our entire patient cohort (830 patients), the most common complication, limb length discrepancy, was reported in only 10 patients. In most cases, the limb length discrepancies were minor and did not result in functional impairment or require additional intervention. While the low incidence suggests that this complication is not a major concern for most patients, it underscores the importance of a careful intraoperative technique and postoperative monitoring to identify and address any clinically significant discrepancies early. One concern was the lack of articles describing postoperative displacement. While complete at final follow-up was reported across many studies, with promising results, the potential for residue malalignment or displacement is still possible [33]. As a postoperative displacement of greater than 2 mm could lead to persistent pain and the potential for early degenerative changes [23,56,57], future research should aim to report this metric more frequently. While this complication was only reported in 11 patients, less than 10% of the included articles described observed rates of this postoperative fracture displacement. As proper anatomic alignment is crucial for optimal long-term success [58], the reporting of postoperative residual displacement should be included in patient outcome series with regularity.

In the articles included in this analysis, outcomes pertaining to conservative treatment were rarely reported. While this may have been a result of our patient cohort being comprised of more complicated cases, future research on clinical outcomes for conservative treatment methods in large patient cohorts should be considered. Importantly, while truly conservative approaches are non-operative (like closed reduction and casting), less invasive surgical options, such as closed reduction with percutaneous fixation, may also be considered depending on the clinical context. As this approach has demonstrated satisfactory mid- to long-term functional outcomes and range of motion retention, cost benefits, and decreased complications compared to anatomic reduction and open techniques [38,59], conservative treatment should only be considered for moderate cases.

It is important to acknowledge the limitations of this study. To begin, no meta-analysis could be performed due to the lack of comparative outcomes and the heterogeneity of the data. Future updates on triplane ankle fractures should aim to perform meta-analyses, particularly on different treatment techniques, diagnostic tools, and complication rates. Next, the overall quality of the included articles was moderate. Future research should aim to study this pathology using more robust research methodologies, including randomized clinical trials. Furthermore, while most clinical outcomes were positive, longer follow-up periods may provide a more comprehensive understanding of this pathology, particularly considering the potential growth and development implications that may come with triplane ankle fractures.

## 5. Conclusions

This review highlights several key aspects of the management of triplane fractures and provides recommendations for future practice and research. Accurate classification is essential for guiding treatment decisions, and the increased use of CT is strongly recommended to better assess fracture complexity, displacement, and articular involvement. While surgical intervention remains the most common approach for triplane fractures, non-operative management should be considered for select cases, such as two-part minimally displaced fractures, under close monitoring.

Limb length discrepancy, the most frequently reported complication, was observed in only 1.2% of patients. While the clinical significance of these discrepancies is uncertain, the low incidence suggests that they are not a major concern for most patients. However, attention to intraoperative techniques and careful follow-up is essential to minimize this risk.

Finally, there is a need for future research to evaluate the comparative outcomes of conservative and surgical treatments for triplane fractures. Long-term studies focusing on functional outcomes, complication rates, and patient satisfaction will be invaluable in optimizing treatment strategies and improving patient care.

## Figures and Tables

**Table 1:** The Methodological Index for Non-Randomized Studies (MINORS) results for each study.

Author (Year)	Total MINORS Score	Clearly Stated Aim	Inclusion of Consecutive Paitents	Prospective Collection of Data	End Points Appropriate to Study Aim	Unbiased Assessment of Study End Point	Follow-Up Period Appropriate to Study Aim	Less than 5% Lost to Follow Up	Prospective Calculation of the Study Size
Aslam (2004) [16]	7	1	1	0	2	0	1	2	0
Ayas (2021) [17]	9	2	1	0	2	0	2	2	0
Brown (2004) [18]	8	2	1	0	1	0	2	2	0
Bund (2020) [19]	7	1	1	0	2	0	1	2	0
Choudhry (2014) [20]	9	2	1	0	2	0	2	2	0
Clement (1987) [21]	7	1	1	0	1	0	2	2	0
Cone (1984) [22]	8	2	1	0	2	0	1	2	0
Cooperman (1978) [23]	10	2	2	0	2	0	2	2	0
Dias (1983) [24]	8	1	1	0	2	0	2	2	0
El-karef (2000) [25]	9	2	1	0	2	0	2	2	0
Ertl (1988) [26]	9	2	1	0	2	0	2	2	0
Gourineni (2011) [27]	10	2	2	0	2	0	2	2	0
Greenhill (2024) [28]	11	2	2	0	2	2	1	2	0
Hadad (2018) [29]	8	2	1	0	1	0	2	2	0
Holland (2018) [30]	8	2	1	0	2	0	1	2	0
Jarvis (2001) [31]	7	1	1	0	1	0	2	2	0
Jennings (2007) [32]	10	2	2	0	2	0	2	2	0
Kim (2009) [33]	9	2	1	0	2	0	2	2	0
Landin (1986) [34]	8	2	1	0	1	0	2	2	0
Ma (2022) [35]	8	2	1	0	2	0	1	2	0
McGillion (2007) [36]	8	1	1	0	2	0	2	2	0
Mishra (2020) [37]	8	2	1	0	2	0	1	2	0
Onay (2023) [38]	9	2	1	0	2	0	2	2	0
Park (2019) [39]	10	2	1	0	2	1	2	2	0
Prijs (2023) [40]	11	2	2	0	2	1	2	2	0
Rapariz (1996) [41]	7	2	1	0	1	0	1	2	0
Ryu (2018) [42]	10	2	2	0	2	0	2	2	0
Seifert (2003) [43]	9	1	2	0	2	0	2	2	0
Tan (2013) [44]	8	2	1	0	2	0	1	2	0
Tang (2022) [45]	9	2	1	0	2	0	2	2	0
Valenza (2021) [46]	7	1	1	0	1	0	2	2	0
van Laarhoven (1995) [47]	9	2	2	0	1	0	2	2	0
Yang (2022) [48]	10	2	1	0	2	1	2	2	0
Zhao (2017) [49]	9	2	1	0	2	0	2	2	0

**Table 2:** Detailed information on study-specific demographics and patient characteristics.

Author (Year)	Number of Patients Who Had Triplane Fractures (Percentage)	% Female	Age in Years (SD/IQR)	Description of Cohorts	Location of Fracture		Mean Follow-Up (Months)
					Right Ankle	Left Ankle	
Aslam (2004) [16]	3 (30)	66.7	12.67 (1.25)	The cohort consists of 10 children aged 8–14 with scooter-related limb injuries	0	3 (100)	6
Ayas (2021) [17]	25 (100)	36	11.6 (2.16)	The cohort consists of 25 patients aged 8–16 years with distal tibia triplane fractures	13 (52)	12 (48)	34.6
Brown (2004) [18]	51 (100)	58.82	13.46 (11.36–15.85)	The study cohort consists of 51 children with tibial triplane fractures	-	-	-
Bund (2020) [19]	16 (42.11)	31.25	14.1 (0.59)	The cohort consists of 38 patients with distal tibia articular fractures, treated using closed reduction	4 (25)	12 (75)	7.44
Choudhry (2014) [20]	58 (74.36)	39.66	13.44	The cohort consists of 78 adolescents with triplane or Tillaux fractures treated surgically	-	-	54
Clement (1987) [21]	15 (100)	53.3	13.67 (11.92–16.33)	The cohort consists of 15 children with triplane fractures who experienced twisting injuries	10 (66.67)	5 (33.33)	19
Cone (1984) [22]	6 (100)	50	13.5 (12–15)	The cohort consists of six adolescents with triplane fractures from accidents like skateboarding and motor vehicle crashes	-	-	-
Cooperman (1978) [23]	15 (100)	60	13.42 (10–16)	The cohort consists of consists of 15 adolescents with triplane fractures, evaluated for treatment and prognosis	-	-	26
Dias (1983) [24]	8 (47.06)	75	8 (1.41)	The cohort consists of 17 adolescents with distal tibial epiphysis fractures, including juvenile Tillaux and triplane fractures, aged 12–16	-	-	27
El-karef (2000) [25]	21 (100)	33.33	13.07 (10.5–15.3)	The cohort consists of consists of 21 patients with triplane fractures of the distal tibia	9 (42.9)	12 (57.14)	28
Ertl (1988) [26]	23 (100)	39.13	13.66 (10.92–16.67)	The cohort consists of 23 patients presenting triplane fractures of the distal tibia due to sports-related injuries	-	-	24
Gourineni (2011) [27]	14 (63.64)	-	14 (1.39)	The cohort consists of 22 skeletally immature patients with displaced Tillaux and triplane fractures	-	-	24
Greenhill (2024) [28]	87 (100)	36.78	12.9 (9.8–15.9)	The cohort consists of 87 pediatric patients, predominantly male, with triplane fractures	-	-	5
Hadad (2018) [29]	33 (100)	39.4	13.59 (0.78)	The cohort consists of 33 children with triplane fractures, studied for fracture pattern mapping	16 (48.48)	17 (51.52)	-
Holland (2018) [30]	5 (100)	40	13.71 (1.59)	The cohort consists of five children with distal tibial triplane fractures and ipsilateral tibial shaft fractures, spanning a period of 56 months	4 (80)	1 (20)	6.8
Jarvis (2001) [31]	6 (100)	16.67	14.05 (1.56)	The cohort consists of six patients with tibial shaft and distal tibial triplane fractures from minor injuries	-	-	22
Jennings (2007) [32]	5 (83.33)	20	13.92 (1.84)	The cohort consists of six patients with juvenile intra-articular epiphyseal ankle fractures treated arthroscopically	4 (80)	1 (20)	27.6
Kim (2009) [33]	11 (78.57)	9.09	13.91 (1.7)	The cohort consists of 14 predominantly male patients treated for triplane and Tillaux fractures	-	-	27.36
Landin (1986) [34]	28 (43.08)	57.14	13.86 (1.8)	The cohort consista of 65 patients with Salter–Harris III and IV lesions, Tillaux, and triplane fractures	-	-	108
Ma (2022) [35]	74 (100)	43.24	14.42 (2.38)	The cohort consists of 74 adolescents with distal tibial triplane fractures treated using cannulated screws or anatomical plates	32 (43.23)	42 (56.76)	6
McGillion (2007) [36]	4 (100)	50	13.5 (0.58)	The cohort consists of four patients with displaced triplane fractures treated with arthroscopy-assisted percutaneous fixation	1 (25)	3 (75)	16
Mishra (2020) [37]	26 (53.06)	26.92	13.4 (1)	The cohort consists of 49 adolescents with transitional distal tibia fractures treated with K-wires or screws	-	-	7.5
Onay (2023) [38]	15 (38.46)	40	13.4 (1.5)	The cohort consists of 39 patients with Salter–Harris II and triplane fractures treated with various surgical techniques	-	-	62.7
Park (2019) [39]	21 (42)	38.1	13.03 (1.53)	The cohort consists of 50 adolescents with displaced Salter–Harris type II, III, or IV tibial fractures	15 (71.43)	6 (28.57)	-
Prijs (2023) [40]	83 (100)	45.78	13.5 (11–18)	The cohort of this mapping study consists of 83 pediatric patients with triplane ankle fractures	55 (66.27)	28 (33.73)	-
Rapariz (1996) [41]	35 (100)	34.29	13 (10–16)	The cohort consists of 35 patients with triplane fractures	20 (57.14)	15 (42.86)	62
Ryu (2018) [42]	33 (100)	36.36	12.69 (1.32)	The cohort consists of 33 patients with triplane fractures treated non-operatively or surgically	-	-	42
Seifert (2003) [43]	5 (22.73)	20	13.8 (0.84)	The cohort consists of 22 adolescents with distal tibial epiphyseal fractures, assessed with MRI	3 (60)	2 (40)	-
Tan (2013) [44]	28 (100)	25	13 (11–15)	The cohort consists of 28 pediatric patients with triplane distal tibial fractures	14 (50)	14 (50)	3.28
Tang (2022) [45]	25 (100)	40	13 (12–15)	The cohort consists of 25 adolescents with triplane fractures treated with lag screws or K-wires	-	-	34
Valenza (2021) [46]	7 (100)	14.29	14 (13–16)	The cohort consists of seven patients with triplane and ipsilateral tibial fractures treated using a novel approach	-	-	78.8
van Laarhoven (1995) [47]	20 (100)	50	-	The cohort consists of 20 adolescents with triplane fractures	-	-	73
Yang (2022) [48]	10 (100)	80	12.3 (11–13)	The cohort consists of 10 adolescents with triplane fractures	5 (50)	5 (50)	10
Zhao (2017) [49]	14 (100)	35.71	14.2 (12–17)	The cohort consists of 14 adolescents with two-part triplane fractures	-	-	15.3

**Table 3:** Details on diagnostic modalities, injury mechanisms, and fracture patterns.

Author (Year)	Imaging Modality	Cause of Fracture (%)						Type of Triplane Fracture (%)			
		Falls/Slips	Scooter/ Skateboard	Sports Activity	Motor Vehicle Accident	Twisting	Unspecified	Two-Part	Three-Part	Four-Part	Five-Part
Aslam (2004) [16]	Radiographs	0	3 (100)	0	0	0	0	-	-	-	-
Ayas (2021) [17]	CT scans	9 (36)	0	9 (36)	7 (28)	0	0	21 (84)	4 (16)	0	0
Brown (2004) [18]	CT scans	0	0	0	0	0	51 (100)	43 (84.31)	8 (15.69)	0	0
Bund (2020) [19]	Radiographs and CT scans	0	0	0	0	0	16 (100)	-	-	-	-
Choudhry (2014) [20]	CT scans	0	0	0	0	0	58 (100)	34 (58.62)	22 (37.93)	2 (3.45)	0
Clement (1987) [21]	Radiographs, conventional tomography, and CT scans	0	0	0	0	15 (100)	0	12 (80)	3 (20)	0	0
Cone (1984) [22]	Radiographs, polytomography, and CT scans	2 (33.3)	2 (33.3)	0	2 (33.3)	0	0	5 (83.33)	1 (16.67)	0	0
Cooperman (1978) [23]	Radiographs, tomography, and CT scans	0	0	0	0	0	15 (100)	-	-	-	-
Dias (1983) [24]	Radiographs, polytomography, and CT scans	0	0	0	0	0	8 (100)	2 (25)	6 (75)	0	0
El-karef (2000) [25]	Radiographs and CT scans	0	0	0	0	21 (100)	0	12 (57.14)	6 (28.57)	3 (14.29)	0
Ertl (1988) [26]	Radiographs, tomography, and CT scans	7 (30.43)	0	15 (65.22)	1 (4.35)	0	0	4 (17.39)	11 (47.83)	0	0
Gourineni (2011) [27]	Radiographs, intraoperative fluorsoscopy, and CT scans	0	0	0	0	0	14 (100)	11 (78.57)	3 (21.43)	0	0
Greenhill (2024) [28]	Radiographs and CT scans	0	0	0	0	0	87 (100)	-	-	-	-
Hadad (2018) [29]	CT scans	0	0	0	0	0	33 (100)	13 (39.39)	14 (42.42)	5 (15.15)	1 (3.03)
Holland (2018) [30]	Radiographs and CT scans	1 (20)	2 (40)	1 (20)	1 (20)	0	0	-	-	-	-
Jarvis (2001) [31]	Radiographs, scanograms, and CT scans	6 (100)	0	0	0	0	0	3 (50)	3 (50)	0	0
Jennings (2007) [32]	Radiographs and CT scans	1 (20)	0	0	1 (20)	3 (60)	0	-	-	-	-
Kim (2009) [33]	Radiographs and CT scans	4 (36.36)	0	5 (45.45)	2 (18.18)	0	0	7 (63.64)	4 (36.36)	0	0
Landin (1986) [34]	Radiographs	0	0	0	0	0	28 (100)	-	-	-	-
Ma (2022) [35]	MRI scans and intraoperative fluoroscopy	14 (18.92)	0	21 (28.38)	39 (52.7)	0	0	-	-	-	-
McGillion (2007) [36]	Radiographs and CT scans	0	0	3 (75)	1 (25)	0	0	-	-	-	-
Mishra (2020) [37]	Radiographs and CT scans	0	0	0	0	0	26 (100)	15 (57.69)	11 (42.31)	0	0
Onay (2023) [38]	Radiographs and CT scans	0	0	0	0	0	15 (100)	-	-	-	-
Park (2019) [39]	MRI and CT scans	0	0	0	0	0	21 (100)	18 (85.71)	3 (14.29)	0	0
Prijs (2023) [40]	CT scans	0	0	0	0	0	83 (100)	55 (66.27)	25 (30.12)	3 (3.61)	0
Rapariz (1996) [41]	Radiographs and CT scans	0	0	17 (48.57)	0	0	18 (51.43)	12 (34.29)	23 (65.71)	0	0
Ryu (2018) [42]	Radiographs and CT scans	15 (45.45)	0	16 (48.48)	2 (6.06)	0	0	29 (87.88)	3 (9.09)	1 (3.03)	0
Seifert (2003) [43]	Radiographs and MRI scans	1 (20)	1 (20)	3 (60)	0	0	0	3 (60)	1 (20)	1 (20)	0
Tan (2013) [44]	Radiographs and CT scans	13 (46.43)	5 (17.86)	10 (35.71)	0	0	0	20 (71.43)	6 (21.43)	2 (7.14)	0
Tang (2022) [45]	Radiographs and CT scans	0	0	0	0	0	25 (100)	-	-	-	-
Valenza (2021) [46]	Radiographs and CT scans	1 (14.29)	0	3 (42.86)	3 (42.86)	0	0	5 (71.43)	2 (28.57)	0	0
van Laarhoven (1995) [47]	Radiographs and CT scans	0	0	15 (75)	1 (5)	0	4 (20)	17 (85)	3 (15)	0	0
Yang (2022) [48]	Radiographs and CT scans	0	0	0	0	0	10 (100)	3 (30)	7 (70)	0	0
Zhao (2017) [49]	Radiographs and CT scans	3 (21.43)	2 (14.29)	9 (64.29)	0	0	0	14 (100)	0	0	0

**Table 4:** Detailed results pertaining to the treatments, additional outcomes, and additional scales.

Author (Year)	Management (%)			Number of Patients with Complete Union at Final Follow-Up (%)	Number of Patients with Fibular Fracture (%)	Number of Patients with Residual Postoperative Displacement > 2 mm (%)	Step-Off on Radiograph in mm (SD)	Scoring Assessment Used at Final Follow-Up		
	Open Reduction With/Without Internal Fixation	Closed Reduction With/Without Percutaneous Osteosynthesis	Conservative Treatment					Modified Weber–Baker score	AOFAS Score	Other Scores
Aslam (2004) [16]	3 (100)	0	0	-	0	-	-	-	-	-
Ayas (2021) [17]	14 (56)	0	11 (44)	-	0	-	-	Excellent: 24/Good: 1/Fair: 0/Poor: 0	99.44	Wong–Baker faces: 0.16
Brown (2004) [18]	-	-	-	34 (66.67)	18 (35.3)	-	-	-	-	-
Bund (2020) [19]	0	16 (100)	0	-	-	-	1.06 (0.61)	Excellent: 13/Good: 3/Fair: 0/: Poor: 0	-	Score of Gleizes: Good: 14/Fair: 2
Choudhry (2014) [20]	4 (6.9)	54 (93.1)	0	-	-	-	-	-	-	-
Clement (1987) [21]	2 (13.33)	4 (26.67)	9 (60)	15 (100)	-	-	-	-	-	-
Cone (1984) [22]	4 (66.67)	2 (33.33)	0	6 (100)	2 (33.3)	-	-	-	-	-
Cooperman (1978) [23]	2 (13.33)	13 (86.67)	0	-	-	-	-	-	-	-
Dias (1983) [24]	3 (37.5)	5 (62.5)	0	8 (100)	3 (37.5)	-	-	-	-	-
El-karef (2000) [25]	7 (33.33)	14 (66.67)	0	21 (100)	5 (23.8)	-	-	-	-	-
Ertl (1988) [26]	7 (30.43)	16 (69.57)	0	-	12 (52.17)	7 (30.43)	-	Excellent: 8/Good: 4/Fair: 2/Poor: 1	-	-
Gourineni (2011) [27]	12 (85.71)	2 (14.29)	0	14 (100)	-	-	-	-	-	-
Greenhill (2024) [28]	65 (74.71)		22 (25.29)	-	-	-	0.2 (0.5)	-	-	-
Hadad (2018) [29]	-	-	-	-	16 (48.48)	-	-	-	-	-
Holland (2018) [30]	1 (20)	3 (60)	1 (20)	5 (100)	3 (60)	-	-	-	-	-
Jarvis (2001) [31]	0	0	6 (100)	6 (100)	-	-	-	-	-	-
Jennings (2007) [32]	5 (100)	0	0	5 (100)	3 (60)	-	-	-	-	-
Kim (2009) [33]	9 (81.82)	2 (18.18)	0	10 (90.91)	6 (54.55)	-	-	Excellent: 10/Good: 0/Fair: 1/Poor: 0	-	-
Landin (1986) [34]	10 (35.71)	0	18 (64.29)	-	10 (35.71)	-	-	-	-	-
Ma (2022) [35]	74 (100)	0	0	67 (90.54)	-	-	-	-	-	Mazur: 90.36
McGillion (2007) [36]	1 (25)	3 (75)	0	4 (100)	-	-	-	-	-	-
Mishra (2020) [37]	26 (100)	0	0	26 (100)	-	-	-	Excellent: 26/Good: 0/Fair: 0/Poor: 0	-	-
Onay (2023) [38]	11 (73.33)	4 (26.67)	0	-	7 (46.67)	-	-	-	94.4	-
Park (2019) [39]	16 (76.19)	1 (4.76)	4 (19.05)	-	15 (71.43)	-	-	-	-	-
Prijs (2023) [40]	-	-	-	74 (89.16)	-	-	-	-	-	-
Rapariz (1996) [41]	20 (57.14)	15 (42.86)	0	-	17 (48.57)	2 (5.71)	-	Excellent: 28/Good: 6/Fair: 1/Poor: 0	-	-
Ryu (2018) [42]	10 (30.3)	4 (12.12)	19 (57.58)	33 (100)	-	2 (6.06)	-	Excellent: 33/Good: 0/Fair: 0/Poor: 0	99.19	-
Seifert (2003) [43]	0	5 (100)	0	5 (100)	-	-	-	-	-	-
Tan (2013) [44]	7 (25)	4 (14.29)	17 (60.71)	28 (100)	11 (35.29)	-	-	Excellent: 28/Good: 0/Fair: 0/Poor: 0	-	-
Tang (2022) [45]	0	25 (100)	0	25 (100)	-	-	-	-	92	-
Valenza (2021) [46]	0	7 (100)	0	7 (100)	-	-	-	-	-	-
van Laarhoven (1995) [47]	9 (45)	1 (5)	10 (50)	-	7 (35)	-	-	-	-	-
Yang (2022) [48]	2 (20)	2 (20)	6 (60)	-	4 (40)	-	-	Excellent: 10/Good: 0/Fair: 0/Poor: 0	-	-
Zhao (2017) [49]	14 (100)	0	0	-	5 (35.71)	-	-	Excellent: 14/Good: 0/Fair: 0/Poor: 0	-	-

**Table 5:** Information on complications from individual articles included in this study.

Author (Year)	Surgical Complications (%)					
	Number of Patients with Leg Length Discrepancy (%)	Implant-Related	Number of Patients with Premature Physis Closure (%)	Infection	Decreased ROM	Other
Aslam (2004) [16]	-	0	-	0	-	0
Ayas (2021) [17]	-	0	-	1 (7.1)	-	0
Brown (2004) [18]	-	-	-	-	-	-
Bund (2020) [19]	-	0	-	0	-	0
Choudhry (2014) [20]	-	-	-	-	-	-
Clement (1987) [21]	-	0	-	0	-	0
Cone (1984) [22]	-	-	-	-	-	-
Cooperman (1978) [23]	-	0	3 (20)	0	1 (6.67)	0
Dias (1983) [24]	-	0	-	0	1 (12.5)	0
El-karef (2000) [25]	5 (23.8)	0	-	0	0	0
Ertl (1988) [26]	1 (4.35)	0	1 (4.35)	0	0	0
Gourineni (2011) [27]	-	1 (7.14)	-	0	0	1 (7.14)
Greenhill (2024) [28]	-	3 (3.45)	2 (2.3)	0	1 (1.15)	0
Hadad (2018) [29]	-	-	-	-	-	-
Holland (2018) [30]	-	-	-	-	-	1 (20)
Jarvis (2001) [31]	3 (50)	0	-	0	0	0
Jennings (2007) [32]	-	-	-	-	-	-
Kim (2009) [33]	-	0	-	0	0	1 (9.09)
Landin (1986) [34]	-	0	-	0	0	0
Ma (2022) [35]	-	4 (5.41)	-	3 (4.05)	0	1 (1.35)
McGillion (2007) [36]	-	-	-	-	-	-
Mishra (2020) [37]	-	0	-	1 (3.85)	0	0
Onay (2023) [38]	-	-	0	-	-	-
Park (2019) [39]	-	0	-	0	0	3 (14.29)
Prijs (2023) [40]	-	-	-	-	-	-
Rapariz (1996) [41]	-	0	-	0	4 (11.43)	2 (5.71)
Ryu (2018) [42]	1 (3.03)	-	-	-	-	-
Seifert (2003) [43]	-	-	1 (20)	-	-	-
Tan (2013) [44]	-	0	-	0	0	0
Tang (2022) [45]	-	-	4 (16)	-	-	-
Valenza (2021) [46]	-	-	-	-	-	-
van Laarhoven (1995) [47]	-	0	-	1 (5)	0	0
Yang (2022) [48]	-	0	-	0	0	1 (10)
Zhao (2017) [49]	-	-	1 (7.14)	-	-	-

## Data Availability

The original contributions presented in this study are included in the article. Further inquiries can be directed to the corresponding author.
